# BioFire^®^ Joint Infection Panel for Samples Other than Synovial Fluid

**DOI:** 10.3390/antibiotics13121198

**Published:** 2024-12-09

**Authors:** Nicola Benvenuto, Stefano Di Bella, Luigi Principe, Diego Luppino, Jacopo Conti, Venera Costantino, Manuela Di Santolo, Marina Busetti, Roberto Luzzati, Verena Zerbato

**Affiliations:** 1 Infectious Diseases Unit, Trieste University Hospital (ASUGI), 34125 Trieste, Italy; 2 Clinical Department of Medical, Surgical and Health Sciences, Trieste University, 34149 Trieste, Italy; 3 Microbiology and Virology Unit, Great Metropolitan Hospital “Bianchi-Melacrino-Morelli”, 89128 Reggio Calabria, Italy; 4 Microbiology Unit, Trieste University Hosital (ASUGI), 34125 Trieste, Italy

**Keywords:** BioFire^®^ Joint Infection Panel, multiplex PCR panels, diagnosis, culture-based diagnosis, diagnostic microbiology

## Abstract

**Objectives**: The early identification of infection-causing microorganisms through multiplex PCR panels enables prompt and targeted antibiotic therapy. This study aimed to assess the performance of the BioFire^®^ Joint Infection Panel (BF-JIP) in analysing non-synovial fluid samples. **Methods**: We conducted a retrospective cohort study at Trieste University Hospital, Italy, on hospitalised adults with non-synovial fluid samples tested by both BF-JIP and traditional culture methods (November 2022–April 2024). **Results**: We evaluated 48 samples from 45 patients, including 24 abscess drainage fluids and 10 tissue samples. The BF-JIP showed high concordance (85.4%) and enhanced detection (4.3%) compared to culture methods. The BF-JIP excelled in cerebrospinal fluid (CSF) (100% accuracy and concordance) and in abscess drainage fluid (accuracy: 95.8%; concordance: 91.7%) identification and maintained high performance rates in patients under antibiotics. **Conclusions**: These findings suggest that BF-JIP is a valuable tool for accurate pathogen detection in various clinical samples, offering the additional advantage of being a rapid method.

## 1. Introduction

Early identification of the microorganisms responsible for infections allows targeted antibiotic therapy to be initiated promptly, which can favourably influence patient outcomes and preserve the efficacy of currently available antibiotics [1,2]. Conventional culture methods, however, can take several hours (or days) and are affected by factors like previous antibiotic treatment, difficult-to-grow pathogens, and poor sample quality, making aetiological identification difficult and often leading to empirical treatment [3,4]. Therefore, culture-independent methods, such as nucleic acid amplification tests, are increasingly being used to diagnose a variety of infectious diseases [1].

Multiplex PCR is a widely used culture-independent method that extends conventional PCR by amplifying multiple target sequences in a single reaction. This technique incorporates several primer pairs, each specific to different targets, allowing for the simultaneous detection of multiple DNA fragments while saving time and resources [5]. The BioFire^®^ FilmArray^®^ system is a multiplex PCR with a total run time of approximately one hour. It simultaneously detects a wide range of pathogens—bacteria, viruses, yeasts, parasites, and antimicrobial resistance genes—through comprehensive panels tailored to specific pathogen groups [6]. Available panels include those for meningitis/encephalitis, gastrointestinal infections, respiratory infections, and sepsis [7]. The BioFire^®^ FilmArray^®^ meningitis/encephalitis panel identifies in cerebrospinal fluid the most common viral, bacterial, and yeast pathogens that cause community-acquired infection in the central nervous system. The BioFire^®^ FilmArray^®^ tests stool samples for 22 common pathogens associated with gastroenteritis. Regarding the respiratory tract infections, there are three panels available—two panels for nasopharyngeal swab samples and one panel (the BioFire^®^ FilmArray^®^ Pneumonia) for samples obtained from sputum, including endotracheal aspirates and bronchoalveolar lavage (BAL), as well as mini-BAL samples. The BioFire^®^ Blood Culture Identification 2 panel detects 43 targets associated with bloodstream infections, including Gram-negative bacteria, Gram-positive bacteria, yeasts, and 10 antimicrobial resistance genes. It provides results in approximately one hour after a positive blood culture [7].

However, for the purpose of this study, we have chosen to focus on the Biofire^®^ Joint Infection Panel (BF-JIP) (bioMérieux, Marcy-l’Etoile, France), which detects 39 targets from synovial fluid obtained from individuals suspected to have a joint infection. Among the targets there are Gram-positive and Gram-negative bacteria, including anaerobes and fungi, and nine resistance genes (Table 1) [8,9]. In a large prospective study conducted in 13 European and American sites, the BF-JIP showed high concordance with traditional culture, along with excellent sensitivity (90.9%) and specificity (98.5%), in detecting joint infections. It also demonstrated a superior turnaround time compared to standard culture systems [9].

This study aimed to evaluate the performance of the BF-JIP in analysing non-synovial fluid samples and compare it with traditional culture methods to identify its potential advantages.

## 2. Methods

### 2.1. Study Design

We conducted a retrospective cohort study at Trieste University Hospital, Italy, including all hospitalised adult patients (aged > 18 years) with at least one non-synovial fluid sample submitted for both culture and BF-JIP testing. The study period ranged from November 2022 to April 2024.

### 2.2. Data Collection

Data collected from electronic medical records included demographics (age, gender), comorbidities, admission date, hospital ward, type of sample analysed, pathogens identified by the BF-JIP and culture, ongoing antimicrobial treatment, and final diagnosis. All data were pseudonymised via a web-based, central, password-protected clinical database management system.

### 2.3. Microbiology

The BF-JIP was used according to the manufacturer’s instructions but for clinical samples other than synovial fluids. Briefly, 200 µL of each sample was pipetted into the provided injection tube. After reconstituting the reaction wells, the sample was applied to the cartridge, and the run started using the BioFire^®^ software. All panels were equipped with all necessary reagents for lysis, nucleic acid extraction, PCR amplification reagents, and internal controls.

Each sample was submitted parallel to traditional culture testing. Bacterial identification was performed by MALDI-TOF mass spectrometry (bioMérieux) and susceptibility tests by the Vitek2 system (bioMérieux). The microbiological culture procedure did not include sonication. For fastidious organisms and anaerobes, we used the disc diffusion method. Susceptibility results were interpreted according to EUCAST criteria.

### 2.4. Data Analysis

To compare the BF-JIP and the culture test (considered the gold standard), the following definitions were employed:

- True Negatives (TN): cases where both methods were negative.
- True Positives (TP): cases with at least one matching microorganism between the BF-JIP and culture.
- False Positives (FP): cases where BF-JIP was positive, but culture was negative.
- False Negatives (FN): cases where BF-JIP was negative, but culture was positive.

A multidisciplinary panel, comprising two Infectious Diseases physicians (V.Z. and S.D.B.) and two microbiologists (L.P. and M.B.), re-evaluated all cases using cultures from alternative specimens and conducted a comprehensive evaluation of the patients’ clinical courses as documented in their medical records.

To assess the performance of the BF-JIP, a suite of statistical metrics was calculated, including percentage of positive agreement (PPA), percentage of negative agreement (NPA), positive predictive value (PPV), negative predictive value (NPV), enhanced detection, concordance (C), and accuracy (A). Concordance was determined by evaluating instances of positive agreement (at least one microorganism detected by both culture and the BF-JIP) and negative agreement (both tests yielding negative results). Accuracy was defined as the proportion of true results (both true positives and true negatives) among the total number of cases examined.

For continuous variables (such as age and the Charlson Comorbidity Index) the median and relative interquartile range (IQR) were calculated.

### 2.5. Ethics Approval

The planning, conduct, and reporting of this study was in line with the Declaration of Helsinki. This study was approved by the Trieste University Ethical Committee (n°V3_2703_24). Consent to participate was assessed according to the Ethical Committee.

## 3. Results

### 3.1. Study Population

A total of 48 samples, corresponding to 45 patients, were included. The cohort comprised predominantly male patients (n = 24), with a median age of 63 years (IQR 45–74) and a median Charlson Comorbidity Index of 3 (IQR 1–5). Most patients were admitted to surgical wards (n = 21). Notably, in 32 cases (67%), patients were undergoing antibiotic therapy at the time of microbiological sample collection. Most of the patients had been diagnosed with a skin and soft-tissue infection (n = 9) or a bone and joint infection (n = 7). The other final diagnoses were abdominal abscesses (n = 6), pleural empyema (n = 5), breast implant infections (n = 5), and meningitis (n = 5). In four cases, infection was ruled out. Population characteristics and final diagnoses are listed in Table 2.

### 3.2. Samples

The samples analysed included 24 abscess drainage fluids (e.g., cerebral, abdominal, skin and soft-tissue abscesses, and infections related to breast implants), 10 biopsies (mainly bone tissue samples collected during surgical procedures), 6 pleural fluid samples, 5 cerebrospinal fluid (CSF) samples, 2 ascitic fluid samples, and 1 vitreous/aqueous humor sample (Table 3).

### 3.3. Microbiology

The BF-JIP detected 42 bacteria and two fungi. Among the Gram-positive bacteria, the most frequently isolated were *Staphylococcus aureus* (n = 6), *Streptococcus* spp. (n = 5), and *Enterococcus faecium* (n = 4). The most frequently isolated among Gram-negatives were *Escherichia coli* (n = 5) and *Haemophilus influenzae* (n = 2). *Candida albicans* was isolated in two samples.

Polymicrobial flora was identified in six cases, all of which matched at least one microorganism from culture. In five out of six of these cases, the BF-JIP identified at least one anaerobic bacterium. Traditional culture did not detect any anaerobes identified by the BF-JIP.

Antimicrobial resistance genes were detected six times. In two cases, resistance profiles were not confirmed by traditional susceptibility tests.

The detailed results of the BF-JIP and standard cultures are shown in Table 4.

**Figure 1:**
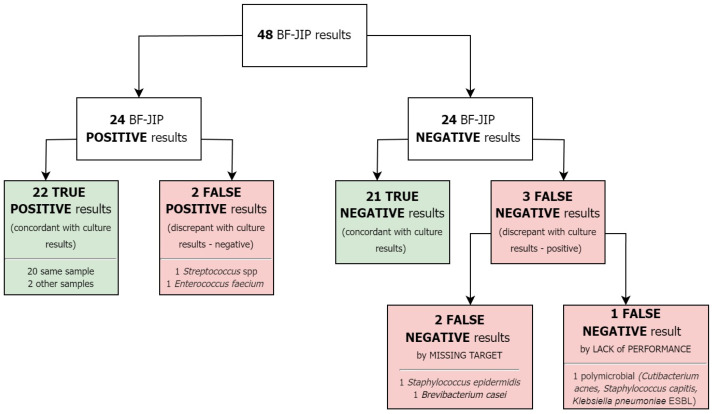
BF-JIP results.

BF-JIP results compared to culture are shown in Figure 1.

In particular, 22 TP, 21 TN, 2 FP, and 3 FN were identified. Two FN were due to pathogens not covered by the panel but identified by traditional culture (*Staphylococcus epidermidis* and *Brevibacterium casei*). The remaining FN was due to the BF-JIP’s lack of performance. Among TP cases, two showed a match between BF-JIP results and cultures from alternative specimens. We reported two FPs: one in a patient without a final diagnosis of infection and the other in a patient with an infection caused by a pathogen not detected by the BF-JIP.

We assessed the BF-JIP performance as follows: concordance of 85.4%, PPA of 88.0%, NPA of 91.3%, PPV of 91.7%, and NPV of 87.5%. The overall accuracy was 89.6%. In addition, we determined that the BF-JIP method had a 4.3% higher detection rate compared to traditional culture methods. Sub-analyses of the most common sample types are detailed in Table 5. The BF-JIP demonstrated its highest performance with cerebrospinal fluid (concordance, accuracy, PPA, NPA, PPV, and NPV of 100%). Abscess drainage fluids were the majority of the analysed samples. For this group, we reported an accuracy of 95.8%, a concordance with culture of 91.7%, a PPA of 100%, an NPA of 90.9%, a PPV of 92.9%, and an NPV of 100%.

A further sub-analysis was conducted to evaluate the BF-JIP performance in patients undergoing antibiotic therapy (n = 32). The concordance with culture was 87.5%. In this context, the BF-JIP showed strong performance with a PPA of 89.5%, NPA of 92.3%, PPV of 94.4%, and NPV of 85.7%.

## 4. Discussion

The BF-JIP demonstrated strong performance in detecting pathogens in non-synovial fluid samples, showing high concordance (85.4%) and enhanced detection (4.3%) when compared to traditional culture methods. In five cases, the panel results did not agree with the culture results, and only one case was due to a missed detection (Figure 1). Parallelly, PPA (88.0%) and overall accuracy (89.6%) were high for the BF-JIP. Our results are consistent with previous studies regarding multiplex PCR panels on synovial fluid, which have confirmed that the BF-JIP has high concordance with traditional culture and excellent sensitivity/PPA and specificity/NPA [9,10,11,12]. It has also been demonstrated that the BF-JIP has a superior turnaround time when compared to standard culture [9,10]. In the setting of joint infections, the BF-JIP established itself as a complementary diagnostic method that can accelerate diagnoses.

Our study is not the first that evaluates the BF-JIP for samples other than synovial fluid. Hoffman et al. evaluated the BF-JIP performance on 23 tissue samples of patients with suspected bone and joint infections, showing a diagnostic power comparable to that of traditional culture [10]. However, the strength of our study lies in the fact that, to our knowledge, it is the first to evaluate the BF-JIP in the context of infections beyond those affecting bone, joint, and soft tissue. Some authors have explored the “off-label” use of other multiplex PCR panels. Micó et al. investigated the use of the blood FilmArray panel on non-blood samples (e.g., cerebrospinal, joint, pleural, ascitic, and abscesses fluids) [13], finding an overall concordance with culture-based methods of 75%. Hirai et al. evaluated the blood FilmArray panel for bone and joint infections [14], showing a sensitivity of 100% when only considering pathogens that are included in the panel. Techniques like 16S rRNA gene next-generation sequencing could also help improve diagnostic capacity in many settings [2], but, unfortunately, they are not available in all centres and, most importantly, do not provide results as quickly as multiplex PCR panels.

Traditional culture did not detect any anaerobes identified by the BF-JIP. Given the increase in antibiotic resistance among anaerobic bacteria and the frequent difficulty in performing susceptibility testing [15], we can consider possible applications of the panel when an anaerobic pathogen is suspected as the cause of infection.

Considering the types of samples analysed, abscess drainage fluids were the most represented. In this group, concordance and accuracy, as well as PPA, NPA, and NPV, were higher compared to the overall group. This is consistent with previous findings and is likely due to the higher bacterial load present in abscesses compared to, for example, ascitic [16] or pleural fluid [13,16]. The best performance of the BF-JIP was observed with cerebrospinal fluid, particularly in five cases of meningitis, three of which were post-surgical. In two cases, the BF-JIP detected the pathogen before the standard culture, helping clinicians to promptly initiate the correct antibiotic therapy. Interestingly, in both cases, the BF-JIP was performed after the multiplex PCR panel for meningitis/encephalitis returned negative results. Given the small sample size, the use of the BF-JIP in this setting warrants further investigation.

We performed an additional sub-analysis in order to evaluate the BF-JIP performance in patients undergoing antibiotic therapy. The performance remained high, but the panel did not show clear superiority over traditional culture. This result may have been affected by the small sample size and sample heterogeneity. Additionally, we did not stratify for the number of days of therapy prior to sampling.

Our study has limitations. First, it is a single-centre study with a relatively small sample size. Second, it is retrospective. Another limitation is the relatively poor variety of samples tested. Third, the BF-JIP is generally associated with high costs. However, no cost-effectiveness analysis comparing this method to traditional culture techniques were conducted in our study. The last and most significant limitation is the lack of a comprehensive assessment of the real clinical impact of the BF-JIP on antibiotic treatment decisions. This gap makes it difficult to draw definitive conclusions about its practical value in guiding therapy. Collectively, these factors limit the generalizability of our results. Future research with larger sample sizes and comprehensive cost-effectiveness analyses will be essential to further validate and optimise this approach.

## 5. Conclusions

The BF-JIP demonstrated strong performance in detecting pathogens in non-synovial fluid samples, showing high concordance with traditional culture methods. Particularly notable was its effectiveness in identifying pathogens in abscess drainage fluids. Additionally, the BF-JIP maintained high performance rates in patients undergoing antibiotic therapy. These findings suggest that the BF-JIP can be a valuable tool for accurate and rapid pathogen detection across a variety of clinical samples, enabling timely and targeted therapies. By facilitating more precise antimicrobial treatments, this approach has the potential to reduce the overuse of broad-spectrum antibiotics, thereby playing a crucial role in preventing the emergence and spread of multidrug-resistant microorganisms. Further studies are warranted to confirm our findings.

## Figures and Tables

**Table 1:** Biofire^®^ Joint Infection Panel (BF-JIP).

Microorganisms Detected by BF-JIP		
Gram +	Gram −	Yeasts
*Anaerococcus prevotii*/*vaginalis*	*Bacteroides fragilis*	*Candida*
*Clostridium perfringens*	*Citrobacter*	*Candida albicans*
*Cutibacterium avidum*-*granulosum*	*Enterobacter cloacae* complex	
*Enterococcus faecalis*	*Escherichia coli*	
*Enterococcus faecium*	*Haemophilus influenzae*	
*Finegoldia magna*	*Kingella kingae*	
*Parvimonas micra*	*Klebsiella aerogenes*	
*Peptoniphilus*	*Klebsiella pneumonia* group	
*Peptostreptococcus anaerobius*	*Morganella morganii*	
*Staphylococcus aureus*	*Neisseria gonorrhoeae*	
*Staphylococcus lugdunensis*	*Proteus* spp.	
*Streptococcus* spp.	*Pseudomonas aeruginosa*	
*Streptococcus agalactiae*	*Salmonella* spp.	
*Streptococcus pneumonia*	*Serratia marcescens*	
*Streptococcus pyogenes*		
Resistance genes: CTX-M, IMP, KPC, mecA/C and MREJ (MRSA), NDM, OXA-48 like, vanA/B, VIM		

CTX-M = cefotaximase-Munich; IMP = Imipenemase; KPC = *Klebsiella pneumoniae* carbapenemase; MRSA = methicillin-resistant *Staphylococcus aureus*; NDM = New-Delhi metallo-β-lactamase; OXA = oxacillinase; VIM = Verona integron-encoded metallo-β-lactamase.

**Table 2:** Population characteristics.

Population Characteristics	
N° samples	48
N° patients	45
Age (median)	63 (IQR 45–74)
Sex	
Male	24 (53%)
Female	21 (47%)
Setting	
Surgical ward	21 (44%)
Medical ward	14 (29%)
ER	6 (13%)
Outpatients	6 (13%)
ICU	1 (<1%)
Antimicrobial therapy	
Yes	32 (67%)
No	15 (33%)
Unknown	1 (<1%)
CCI (median)	3 (IQR 1–5)
Final diagnosis	
SSTI	9 (19%)
BJI	7 (15%)
Abdominal abscess	6 (13%)
Pleural empyema	5 (10%)
Breast implant infection	5 (10%)
Meningitis	5 (10%)
No evidence of infection	4 (8%)
Others	7 (15%)

BJI = bone and joint infection; CCI = Charlson Comorbidity Index; ER = emergency room; ICU = intensive care unit; IQR = interquartile range; SSTI = skin and soft-tissue infection.

**Table 3:** Samples on which the BF-JIP was performed.

Sample Types	
Abscess drainage fluid	24 (50%)
Biopsy	10 (21%)
Pleural fluid	6 (13%)
CSF	5 (10%)
Ascitic fluid	2 (<1%)
Vitreous humor/aqueous humor	1 (<1%)

CSF = cerebrospinal fluid.

**Table 4:** Comparison between cultures and the BF-JIP.

BF-JIP and Culture Comparison					
N°	Sample	BF-JIP	Culture (Same Sample)	Other Cultures	Final Diagnosis
1	Biopsy	*Parvimonas micra*; *Peptostreptococcus anaerobius*; *Streptococcus* spp.	*Streptococcus anginosus*		SSTI
2	Abscess drainage liquid	*Staphylococcus aureus* mecA/B and MREJ	*Staphylococcus aureus*		Brain abscess
3	Ascitic fluid				Peritonitis
4	Abscess drainage liquid	*Streptococcus* spp.			No infection
5	Abscess drainage liquid	*Escherichia coli*	*Escherichia coli*		BJI
6	Abscess drainage liquid	*Anaerococcus prevotii*; *Finegoldia magna*; *Peptoniphilus*; *Proteus* spp.	*Proteus mirabilis*		SSTI
7	Abscess drainage liquid	*Staphylococcus aureus*	*Staphylococcus aureus*		BJI
8	Pleural fluid				Pulmonary aspergillosis
9	Pleural fluid	*Streptococcus pyogenes*	*Streptococcus pyogenes*		Pleural empyema
10	Ascitic fluid	*Enterococcus faecium*; *Candida albicans* VanA/B	*Candida albicans*		Peritonitis
11	Pleural fluid	*Staphylococcus aureus*		*Staphylococcus aureus* (blood culture and BAL)	Pleural empyema
12	Abscess drainage liquid	*Escherichia coli*; *Peptoniphilus*	*Escherichia coli*		Abdominal abscess
13	Pleural fluid				Pleural empyema
14	Biopsy		*Staphylococcus* *epidermidis*		SSTI
15	Abscess drainage liquid				Abdominal abscess
16	CSF				Meningitis
17	Abscess drainage liquid	*Anaerococcus prevotii*; *Enterococcus faecium*; *Finegoldia magna*; *Parvimonas micra*; *Streptococcus* spp.; *Bacteroides fragilis*; *Citrobacter*; *Enterobacter cloacae*; *Escherichia coli*; *Haemophilus influenzae*; *Klebsiella pneumoniae*; *Candida albicans* CTX-M, vanA/B, VIM	*Pseudomonas aeruginosa*; *Klebsiella pneumoniae*; *Candida albicans*		Abdominal abscess
18	Biopsy	*Pseudomonas aeruginosa*	*Pseudomonas aeruginosa*		SSTI
19	Pleural fluid	*Enterococcus faecium*			Pleural empyema
20	Biopsy	*Bacteroides fragilis*	*Bacteroides fragilis*		SSTI
21	Abscess drainage liquid	*Escherichia coli*	*Escherichia coli*		Abdominal abscess
22	Abscess drainage liquid	*Streptococcus pyogenes*	*Streptococcus pyogenes*		Necrotizing fasciitis
23	Abscess drainage liquid	*Staphylococcus aureus*	*Staphylococcus aureus*		Muscle haematoma
24	Abscess drainage liquid				SSTI
25	Abscess drainage liquid				No infection
26	Biopsy				BJI
27	Abscess drainage liquid	*Escherichia coli*		*Candida glabrata* (abscess drainage fluid and blood culture); *Escherichia coli* (blood culture); *Pseudomonas aeruginosa* (abscess drainage fluid); *Klebsiella pneumoniae* (abscess-drainage fluid)	Abdominal abscess
28	Abscess drainage liquid				BJI
29	Abscess drainage liquid				No infection
30	Abscess drainage liquid				Abdominal abscess
31	CSF				Meningitis
32	vitreous humor/aqueous humor				Endophthalmitis
33	CSF	*Streptococcus* spp.	*Streptococcus salivarius*		Meningitis
34	Biopsy		*Cutibacterium acnes*; *Staphylococcus capitis*; *Klebsiella pneumoniae*		BJI
35	Biopsy	*Enterococcus faecalis*	*Enterococcus faecalis*		SSTI
36	Pleural fluid				Pleural empyema
37	Abscess drainage liquid				Breast implant infection
38	Abscess drainage liquid				Breast implant infection
39	CSF				Meningitis
40	Abscess drainage liquid	*Parvimonas micra*; *Streptococcus* spp.	*Streptococcus anginosus*		SSTI
41	Abscess drainage liquid				No infection
42	Abscess drainage liquid	*Staphylococcus aureus*	*Staphylococcus aureus*		Breast implant infection
43	Abscess drainage liquid	*Staphylococcus aureus*	*Staphylococcus aureus*		SSTI
44	Biopsy				BJI
45	CSF	*Haemophilus influenzae*	*Haemophilus influenzae*		Meningitis
46	Biopsy				BJI
47	Biopsy	*Enterococcus faecium*	*Brevibacterium casei*		Breast implant infection
48	Abscess drainage liquid	*Parvimonas micra*; *Peptostreptococcus anaerobius*; *Streptococcus* spp.			Breast implant infection

BAL = broncho-alveolar lavage; BJI = bone and joint infection; CSF = cerebrospinal fluid; CTX-M = cefotaximase-Munich; MREJ = methicillin resistance-encoding gene junction; SSTI = skin and soft-tissue infection; VIM = Verona integron-encoded metallo-β-lactamase.

**Table 5:** Performances of the BF-JIP.

BF-JIP Performances						
	PPA	NPA	PPV	NPV	C	A
All samples (n = 48)	88.0%	91.3%	91.7%	87.5%	85.4%	89.6%
Abscess drainage fluid (n = 24)	100%	90.9%	92.9%	100%	91.7%	95.8%
Biopsy (n = 10)	57.1%	100%	100%	50%	70.0%	70.0%
Pleural fluid (n = 6)	100%	75%	66.7%	100%	66.7%	83.3%
Cerebrospinal fluid (n = 5)	100%	100%	100%	100%	100%	100%

A = accuracy; C = concordance; NPA = percentage of negative agreement; NPV = negative predictive value; PPA = percentage of positive agreement; PPV = positive predictive value.

## Data Availability

The datasets used and/or analysed during this current study are available from the corresponding author on reasonable request.

## Abbreviations

BF-JIP	BioFire^®^ Joint Infection Panel
CSF	Cerebrospinal fluid
TN	True Negatives
TP	True Positives
FP	False Positives
FN	False Negatives
A	Accuracy
C	Concordance
NPA	Percentage of Negative Agreement
NPV	Negative Predictive Value
PPA	Percentage of Positive Agreement
PPV	Positive Predictive Value
